# New records of tick-host mammal interactions (Acari: Ixodidae) in two conservation units of the Brazilian Amazon

**DOI:** 10.1590/S1984-29612026014

**Published:** 2026-06-15

**Authors:** Ivaneide Nunes da Costa, Marcos Valério Garcia, Lunna Cunha Silva Aguirre, Gabriel Moreira Valença, Vanessa Paiva dos Santos, Natalia Vitória Coelho Costa, Angélica Lorena Pereira Mendes Carioca, Paulo Sergio D’Andrea, Renato Andreotti, Jansen Fernandes Medeiros, André de Abreu Rangel Aguirre

**Affiliations:** 1 Fundação Oswaldo Cruz – Fiocruz Rondônia, Laboratório de Entomologia, Porto Velho, RO, Brasil; 2 Fundação Oswaldo Cruz – Fiocruz Rondônia, Instituto Oswaldo Cruz, Programa de Pós-graduação em Biologia Parasitária, Porto Velho, RO, Brasil; 3 Embrapa Gado de Corte, Laboratório de Biologia do Carrapato, Campo Grande, MS, Brasil; 4 Universidade Federal de Rondônia, Programa de Pós-graduação em Biologia Experimental – PGBIOEXP, Porto Velho, RO, Brasil; 5 Fundação Oswaldo Cruz – Fiocruz, Instituto Oswaldo Cruz, Laboratório de Biologia e Parasitologia de Mamíferos Reservatórios, Rio de Janeiro, RJ, Brasil; 6 Embrapa Gado de Corte, Laboratório de Biologia Molecular, Campo Grande, MS, Brasil; 7 Fundação Oswaldo Cruz – Fiocruz Rondônia, Plataforma de Criação e Experimentação Animal, Porto Velho, RO, Brasil

**Keywords:** Wild mammals, Amblyomma, Rondonia, Amazonas, Mamíferos silvestres, Amblyomma, Rondônia, Amazonas

## Abstract

Ticks are ectoparasites of major public and veterinary health importance, and identifying their vertebrate hosts is crucial for recognizing pathogen reservoirs. This study investigated tick–wild mammal associations in two areas of the Brazilian Amazon. Between 2021 and 2023, wild mammals were captured using Tomahawk® and Sherman® traps, and ticks were collected manually or with forceps. A total of 110 wild mammals were captured, including 66 marsupials, 43 rodents, and one felid, representing 12 genera. Three species were identified at the species level: *Didelphis marsupialis*, *Myoprocta pratti*, and *Leopardus pardalis*. Tick infestation was recorded in 37.3% of the animals, with 262 ticks collected. The identified tick species were *Amblyomma coelebs*, *Amblyomma ovale*, *Amblyomma latepunctatum*, *Amblyomma scalpturatum*, *Amblyomma pacae*, and *Haemaphysalis* sp. These findings expand knowledge of tick–host associations involving wild mammals in the Brazilian Amazon.

## Resumo

Os carrapatos são ectoparasitos de grande importância para a saúde pública e veterinária, e o conhecimento sobre seus hospedeiros é essencial para a identificação de reservatórios de patógenos. Este estudo teve como objetivo investigar as associações entre carrapatos e mamíferos silvestres em duas áreas da Amazônia Brasileira. Entre 2021 e 2023, mamíferos silvestres foram capturados com armadilhas Tomahawk® e Sherman®, e os carrapatos foram coletados manualmente ou com o auxílio de pinças. Um total de 110 mamíferos silvestres foi capturado, incluindo 66 marsupiais, 43 roedores e um felídeo, distribuídos em 12 gêneros. Três espécies foram identificadas em nível específico: *Didelphis marsupialis*, *Myoprocta pratti* e *Leopardus pardalis*. A infestação por carrapatos foi registrada em 37,3% dos animais, totalizando 262 ectoparasitos coletados. As espécies de carrapatos identificadas foram *Amblyomma coelebs*, *Amblyomma ovale*, *Amblyomma latepunctatum*, *Amblyomma scalpturatum*, *Amblyomma pacae* e *Haemaphysalis* sp. Os resultados ampliam o conhecimento sobre as interações carrapato–hospedeiro envolvendo mamíferos silvestres na região amazônica.

Ticks (Acari: Ixodida) are obligate ectoparasites that parasitize various animal groups. In the Americas, the main genera found on wild mammals are *Amblyomma*, *Ixodes*, *Rhipicephalus*, *Dermacentor* and *Haemaphysalis* (Guglielmone et al., 2021). *Amblyomma* is the most diverse genus in the Neotropical region and parasitizes a wide range of hosts, including mammals, birds, reptiles and amphibians (Guglielmone et al., 2021). In Brazil, most tick species recorded in the literature parasitize wild mammals. In the Amazon, a high diversity of ticks has been reported on rodents, marsupials, suids and carnivores, and mammals play an important role in the maintenance and dispersion of ticks in preserved and anthropized ecosystems (Luz et al., 2021).

Despite the high diversity of tick species parasitizing wild animals, gaps remain in the knowledge of local wild mammal fauna and their interactions with ticks. Understanding tick diversity associated with wild mammals is essential for identifying potential hosts and species of vector importance. In this context, this study reports new associations between ticks and wild hosts in the Brazilian Amazon, expanding knowledge of tick–host relationships in the region.

**Figure 1:**
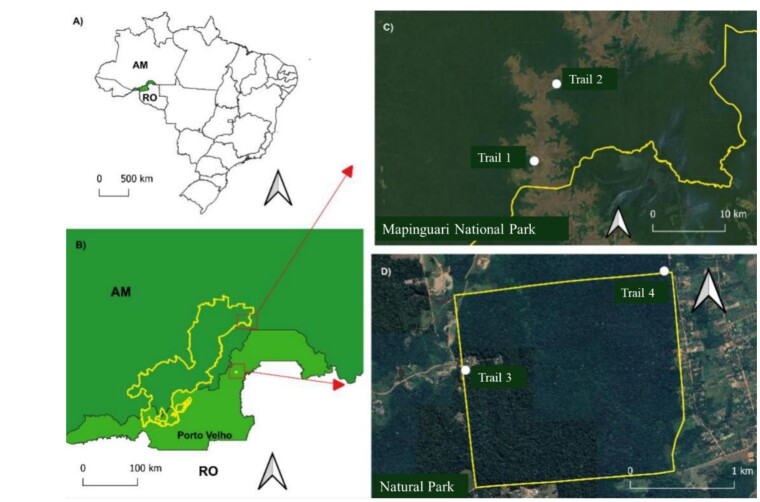
Map of Brazil highlighting the states of Rondônia and Amazonas (A). State of Amazonas and the municipality of Porto Velho highlighting the areas of PARNA Mapinguari and Natural Park de Porto Velho (B). (C) PARNA Mapinguari area showing the mammal collection points (Trail 1 and 2). (D) Natural Park area showing the collection points (Trail 3 and 4).

This study was conducted in two conservation units (CUs): Mapinguari National Park (MNP) in Canutama, Amazonas (7º51’45.3” S 63º51’30.3” W) and the Porto Velho Municipal Natural Park (NP) in Porto Velho, Rondônia (8º41’12.9” S 63º52’02.8” W) (Figure 1).

Wild animal collections were conducted over two years, with four campaigns per area (two in the dry and two in the rainy season) along four trails in MNP and NP. Medium-sized mammals were captured using large Tomahawk traps baited with animal and plant-based food, while small mammals were captured using transects combining Sherman® and small Tomahawk® traps placed at ground level and in the understory. Traps were spaced 15 m apart, transects 500 m apart, baited with a mixed attractant, and set for seven consecutive nights.

Captured animals were taken to an NB3 field laboratory following Oswaldo Cruz Institute biosafety recommendations (Lemos & D’Andrea, 2014). Rodents and marsupials were anesthetized with ketamine, xylazine and butorphanol and euthanized by cardiac exsanguination, while felids were sedated with ketamine, midazolam and butorphanol, with sedation reversed using flumazenil. Euthanasia was restricted to rodents and marsupials for integrative taxonomy and related studies. Animals were identified at the genus level based on external morphology, and rodent species through integrative taxonomy (Faria et al., 2019; Bonvicino et al., 2008). Collections were authorised by ICMBio (licence No. 71739-10) and approved by the Ethics Committee on the Use of Animals of the Oswaldo Cruz Institute (protocol No. 2019/21).

Immediately after the procedures described above, animals were inspected and ticks were removed using the TickLasso (Silvalure®, Laholm, Sweden), tweezers or manually. Engorged immature stages (larvae and nymphs) were collected alive, stored in plastic containers and maintained in an incubator at 23–28 °C, 70–85% relative humidity and a 12 h light/12 h dark photoperiod until molting (Pacheco et al., 2021).

Tick identification was performed using a stereoscopic microscope and dichotomous keys for Ixodida families and genera (modified by Dantas-Torres et al., 2019) and for *Amblyomma* nymphs (Martins et al., 2010). Larvae and damaged nymphs were identified by sequencing fragments of the 16S rRNA gene (460 bp) (Mangold et al., 1998) and the cytochrome oxidase subunit I (COI) gene (710 bp) (Folmer et al., 1994)

A total of 110 wild mammals were captured in the two study areas, of which 41 (37.3%) were infested with ticks. The captures included 66 marsupials, 43 rodents and one felid carnivore, representing 12 mammal genera. Marsupials (Didelphidae) comprised (Didelphidae): *Didelphis marsupialis* Linnaeus, 1758 (47 individuals collected), *Marmosa* sp. (15), *Marmosops* sp. (01), *Metachirus* sp. (01), *Philander* sp. (01) and *Caluromys* sp. (01). Five rodent genera were identified: *Proechimys* sp. (31) and *Mesomys* sp. (03) (Rodentia: Echimyidae); *Oecomys* sp. (07) and *Necromys* sp. (01) (Rodentia: Sigmodontinae); and *Myoprocta pratti* Pocock, 1913 (01) (Rodentia, Dasyproctidae). A single felid, *Leopardus pardalis* Linnaeus, 1758 (Felidae), was also recorded (Table 1).

**Table 1:** Wild mammals sampled in PARNA Mapinguari (MNP), AM and Natural Park, RO (NP).

**Animals captured**	**Location**			**Total**
	**Mapinguari National Park**	**Natural Park**		
**Marsupials**				
*Didelphis marsupialis*	20	27	47	
*Marmosa* sp.	15		15	
*Marmosops* sp.	1		1	
*Caluromys* sp.	0	1	1	
*Metachirus* sp.	0	1	1	
*Philander* sp.	1		1	
**Rodents**				
*Proechimys* sp.	15	16	31	
*Oecomys* sp.	2	5	7	
*Mesomys* sp.	3	0	3	
*Necromys* sp.	1	0	1	
*Myoprocta pratti*	1	0	1	
**Felids**				
*Leopardus pardalis*	1	0	1	
TOTAL	60	50	110	

In MNP, tick infestation prevalence was 33.3% (20/60), whereas in NP it was 42.0% (21/50). A total of 262 immature ticks (126 nymphs and 136 larvae) were collected from 41 animals (Table 2). In MNP, 80% of *D. marsupialis* (16/20) were infested. The identified species were *Amblyomma coelebs* Neumann, 1899 (one larva molecularly identified by the 16S rRNA gene, 99% similarity with GenBank MN065775.1, and 35 nymphs identified taxonomically), *Amblyomma latepunctatum* Tonelli-Rondelli, 1939 (40 nymphs identified taxonomically and one nymph molecularly confirmed by the COI gene, 95% similarity with GenBank MH513228.1), and one nymph of *Amblyomma scalpturatum* Neumann, 1906. Additionally, 64 larvae and nine nymphs collected from *D. marsupialis* in MNP could not be identified to species level due to lack of reliable PCR amplicons and were classified as *Amblyomma* sp.

**Table 2:** Ticks collected from wild mammals in PARNA Mapinguari (MNP), AM and Natural Park, RO (NP).

**Location/species of wild mammals** ^*^ **(No. of infested animals/No. of individuals captured)**	**Stages**	
**Tick species**	**Larvae**	**Nymph**
MNP/*Didelphis marsupialis* (16/20)		
*Amblyomma latepunctatum*		41
*Amblyomma coelebs*	1	35
*Amblyomma scalpturatum*		1
*Amblyomma* sp.	64	9
		
MNP*/Philander* sp*.* (1/1)		
*Amblyomma* sp*.*	1	
		
MNP/*Myoprocta pratti* (1/1)		
*Amblyomma naponense*		7
*Amblyomma* sp.	1	
		
MNP*/Proechimys* sp*.* (1/15)		
*Amblyomma coelebs*	1	
		
MNP/*Leopardus pardalis* (1/1)		
*Amblyomma scalpturatum*		1
		
NP*/Didelphis marsupialis* (14/27)		
*Amblyomma ovale*	31	4
*Amblyomma coelebs*		16
*Amblyomma pacae*		5
*Amblyomma varium*	1	
*Haemaphysalis* sp.		1
*Amblyomma* sp*.*	35	
		
NP*/Metachirus* sp*.* (1/1)		
*Amblyomma pacae*		1
		
NP*/Proechimys* sp*.* (6/16)		
*Amblyomma ovale*		5
*Amblyomma* sp*.*	1	

* Only the animals infested with ticks were included.

One tick larva was collected from *Proechimys* sp. (6.7%; 1/15) and molecularly identified as *A. coelebs* by the 16S rRNA gene, showing 99% similarity with GenBank MH513258.1. Another unidentified larva collected from *Philander* sp. was classified as *Amblyomma* sp. From *M. pratti*, eight ticks were collected, including seven nymphs of *Amblyomma naponense* (Packard, 1869) and one larva identified as *Amblyomma* sp. From *L. pardalis*, one nymph was molecularly identified by a partial 16S rRNA sequence, showing 98.76% identity (399/404) with *A. scalpturatum* (GenBank MH513291.1).

In NP, 51.9% (14/27) of *D. marsupialis* were infested with ticks. The identified species were *A. coelebs* (16 nymphs), *Amblyomma ovale* Koch, 1844 (four nymphs and 31 engorged larvae that molted into nymphs), and *Amblyomma pacae* Aragão, 1911 (three nymphs identified taxonomically and two by partial 16S rRNA sequencing with 98% identity in GenBank, OQ650187.1). In addition, 36 larvae were collected, including one molecularly identified as *Amblyomma varium* Koch, 1844 (98% 16S rRNA identity, GenBank MH818416.1) and 35 classified as *Amblyomma* sp., as well as one nymph of *Haemaphysalis* sp. In *Proechimys* sp., 37.5% (6/16) were infested, with five nymphs of *A. ovale* and one larva and one nymph classified as *Amblyomma* sp. One individual of *Metachirus* sp. was infested with a single nymph of *A. pacae*, molecularly identified by a partial 16S rRNA sequence showing 98% similarity (404/411) with GenBank accession OQ650187.1.

Tick diversity associated with wild mammals varies according to biogeographic region, habitat type and host availability. In this study, higher tick abundance was observed on *D. marsupialis*, with *A. latepunctatum* and *A. coelebs* being more frequent in MNP, and *A. coelebs* and *A. ovale* in NP, all in immature stages.

In this study, the occurrence of immature stages of *A. latepunctatum* and *A. coelebs* on opossums was expected, as parasitism of these tick species on small wild mammals, including *D. marsupialis*, has been previously reported (Labruna et al., 2005a; Labruna et al., 2010; Guglielmone et al., 2014; Martins et al., 2014; Gianizella et al., 2018; Binetruy et al., 2019). Records of *A. coelebs* nymphs parasitizing *Didelphis albiventris* (Lund, 1840) are also deposited in the National Tick Collection (CNC) (Labruna et al., 2005a). These data reinforce the parasite–host association between *A. latepunctatum*, *A. coelebs* and opossums. In addition, this study reports a new record of *A. coelebs* nymphs parasitizing *Metachirus* sp. (Didelphimorphia).

*Amblyomma ovale* has a wide distribution from the United States to South America. Adult specimens have been reported parasitizing several wild mammals, including *Pecari tajacu* (L., 1758), *Tayassu pecari* Link, 1795), *Dasyprocta* spp., *Cuniculus paca* (L., 1766), *Tapirus terrestris* (L., 1758), *Panthera onca* (L., 1758), *Sus scrofa* (L., 1758), *Eira barbara* (L., 1758) and *Nasua nasua* (L., 1766), as well as dogs, cervids and humans (Labruna et al., 2005a). Although no adult *A. ovale* were recorded on wild mammals in the present study, adults parasitizing dogs in NP were recently reported by our research group (Costa et al., 2025). This study recorded 31 engorged larvae and four nymphs of *A. ovale* parasitizing *D. marsupialis*, and five nymphs parasitizing wild rodents. Immature stages of *A. ovale* are mainly associated with small rodents (Szabó et al., 2013); however, among the 49 rodents captured, only five individuals of *Proechimys* (10.2%; 5/49) were infested, each with a single nymph. These findings agree with previous data from the same region reporting a single *A. ovale* nymph on a rodent (Labruna et al., 2005a). The low prevalence of *A. ovale* on rodents suggests that this tick–host association remains weak in the Amazon, possibly indicating a stronger relationship with marsupials, especially *D. marsupialis*, although further studies are needed.

To date, only one record of *A. ovale* parasitizing *D. marsupialis* has been reported, describing a single nymph in Panama (Domínguez et al., 2019). The present study provides the first record of both larvae and nymphs of *A. ovale* parasitizing *D. marsupialis* in Brazil and reports a 100% molting rate of engorged larvae into nymphs. These findings suggest that *D. marsupialis* may play an important role in maintaining *A. ovale* in nature, particularly in anthropized areas where opossums are common. Due to their synanthropic behaviour and ecological plasticity, opossums frequently move between wild and urban environments, come into contact with multiple hosts, and act as bridges between ecosystems (Malta & Luppi, 2007; Braga et al., 2023). As they are parasitized by several tick species, opossums may contribute to tick dispersion and facilitate interactions among vectors, hosts and humans, reinforcing their potential involvement in spillover processes (Serpa et al., 2021). The detection of *A. ovale* parasitizing *D. marsupialis* in areas close to urbanization (NP) highlights the importance of monitoring synanthropic species and their parasites within integrated health surveillance and zoonosis prevention strategies.

The least abundant tick species recorded were *A. scalpturatum* parasitizing *D. marsupialis* and *L. pardalis*; *A. pacae*, *A. varium* and *Haemaphysalis* sp. on *D. marsupialis*; and *A. naponense* on *M. pratti*. *Amblyomma scalpturatum* is predominantly reported in the Amazon biome, where adult stages preferentially parasitize large mammals, especially *Tapirus terrestris* (Labruna et al., 2005a). Larvae of this species have been recorded parasitizing *D. marsupialis* (Colle et al., 2020), while nymphs have been reported on dogs, domestic pigs and other wild mammals (Labruna et al., 2005b). In the present study, a single nymph of *A. scalpturatum* was found parasitizing *L. pardalis*, representing the first record of this association and expanding knowledge of the host range of this species in the Neotropical region. As noted by Guglielmone et al. (2021), the specific hosts of the immature stages of this tick remain poorly defined, and this study provides an additional parasite–host record for the literature.

*Haemaphysalis* is a genus of ticks belonging to the family Ixodidae with a wide global distribution. Among the 165 described species, only three have been recorded in the Neotropical region: *Haemaphysalis leporispalustris* (Packard, 1869), *Haemaphysalis juxtakochi* Cooley, 1946 and *Haemaphysalis cinnabarina* Koch, 1844). *Haemaphysalis juxtakochi* is commonly found parasitizing cervids, but it can also parasitize other wild and domestic mammals, including dogs (Labruna et al., 2005a). A study conducted in Panama reported larvae and nymphs of *H. juxtakochi* parasitizing *D. marsupialis*, as well as other wild mammals (Domínguez et al., 2019). In the present study, a single specimen identified as *Haemaphysalis* sp. in the nymphal stage was found parasitizing *D. marsupialis*. However, due to its deteriorated condition, identification at the species level and molecular confirmation were not possible. In the state of Rondônia, only *H. juxtakochi* has been recorded to date; therefore, the specimen collected is likely to belong to this species, but it was conservatively identified only at the genus level.

Additionally, *A. varium* is a Neotropical tick species commonly associated with sloths, *Bradypus* spp. and *Choloepus* spp. (Pilosa: Bradypodidae). However, literature reports indicate its occurrence in other vertebrate groups, including marsupials of the genus *Didelphis*, as reported in a study conducted in Panama, in which the authors suggested *D. marsupialis* as the host species (Marques et al., 2002). In Brazil, there are no records to date of parasitism by species of the genera *Haemaphysalis* or *A. varium* in *D. marsupialis*, making this the first report of these tick–host associations in the country.

The species *A. naponense* is widely distributed in the Neotropical region, occurring in Central and South America. Adult stages show a feeding preference for wild suids (Tayassuidae), while immature stages parasitize a wide range of wild mammals (Luz et al., 2021; Guglielmone et al., 2021). Although *A. naponense* has been reported parasitizing species of the genus *Myoprocta*, this study provides the first record of this tick parasitizing *M. pratti*, expanding knowledge of tick–host associations in wild fauna.

The results of this study expand the understanding of parasite–host interactions in Brazil by documenting new associations between tick species and wild mammals. These findings enhance our knowledge of the ecology of ectoparasites in the Amazon region and emphasize the importance of continuous monitoring of tick fauna and their hosts. Such monitoring is essential not only in areas of high biodiversity, such as the Amazon biome, but also in degraded environments subject to anthropogenic expansion, where the presence of synanthropic animals, such as the opossum (*D. marsupialis*), is more frequent. Therefore, this work provides valuable information for future studies on the dynamics of vector and pathogen circulation, contributing to preventive actions in public and veterinary health.

## Data Availability

All data are included in the article.
